# A new insight into ductile fracture of ultrafine-grained Al-Mg alloys

**DOI:** 10.1038/srep09568

**Published:** 2015-04-08

**Authors:** Hailiang Yu, A. Kiet Tieu, Cheng Lu, Xiong Liu, Mao Liu, Ajit Godbole, Charlie Kong, Qinghua Qin

**Affiliations:** 1 School of Mechanical, Materials & Mechatronics Engineering, University of Wollongong, Wollongong, NSW 2500, Australia; 2 Electron Microscope Unit, University of New South Wales, Sydney, NSW 2052, Australia; 3 Research School of Engineering, Australian National University, Canberra, ACT 2601, Australia

## Abstract

It is well known that when coarse-grained metals undergo severe plastic deformation to be transformed into nano-grained metals, their ductility is reduced. However, there are no ductile fracture criteria developed based on grain refinement. In this paper, we propose a new relationship between ductile fracture and grain refinement during deformation, considering factors besides void nucleation and growth. Ultrafine-grained Al-Mg alloy sheets were fabricated using different rolling techniques at room and cryogenic temperatures. It is proposed for the first time that features of the microstructure near the fracture surface can be used to explain the ductile fracture post necking directly. We found that as grains are refined to a nano size which approaches the theoretical minimum achievable value, the material becomes brittle at the shear band zone. This may explain the tendency for ductile fracture in metals under plastic deformation.

Since the brittle fracture model proposed by Griffith in the last century, many studies focusing on fracture mechanics have been carried out^1^,^2^,^3^. However, the exact nature of ductile fracture remains largely unknown, despite the great deal of attention it has received, especially with the development of nano-grained materials^4^,^5^,^6^.

Nano-grained/ultrafine-grained bulk metals produced using severe plastic deformation (SPD) techniques show excellent mechanical properties^7^,^8^,^9^,^10^. SPD techniques such as equal channel angular pressing^11^, high pressure torsion^12^ and accumulative rolling bonding^13^ have been developed in the past 30 years. However, such techniques are rarely used to produce continuous sheets/foils. Sheets/foils are widely used in the aerospace, automotive, marine and other industries, in which they are required to have both excellent ductility and strength. In general, sheets/foils are produced by cold rolling. Examination of the microstructures of cold-rolled sheets/foils has shown that the cold rolling process increases the extent of centerline segregation of the second-phase particles^14^. Compared with cold rolling, the asymmetric rolling (AR) technique is better suited to produce thinner foils due to the formation of a cross-shear region in the rolling deformation zone^15^. Moreover, AR is a continuous SPD technique suitable for achieving a marked reduction in grain size below the micron level^16^. Cryorolling is another SPD technique that can be used to produce nano-grained materials^17^,^18^. Asymmetric cryorolling (ACR) is a technique that combines the features of AR and cryorolling, and has been used to produce nano-grained aluminum sheets^19^,^20^. Generally, the techniques used to obtain an ultrafine-grained microstructure strongly influence the mechanical properties of the materials^21^. This is primarily because different techniques result in differences in the microstructure, such as distribution of grains and the presence of subgrains, dislocation density and boundary characteristics.

The ultrafine grains and the high density of grain boundaries restrict the nucleation, and also the movement and the interaction of dislocations^22^. Although many deformation mechanisms such as grain rotation^23^ and grain boundary sliding^24^ have been proposed to explain the improvement in ductility of nano-grained materials, many nano-grained materials exhibit very low ductility and very limited strain hardening. As described by Ritchie^25^, in the quest for stronger and harder materials, bulk structural materials without an appropriate fracture resistance are of limited benefit. In contrast, most coarse-grained metals subjected to tensile tests are tested to ductile fracture. Many ductile fracture criteria have been proposed. The basic principles upon which ductile fracture criteria are based can be generally divided into four categories^26^: (a) energy dissipation; (b) void growth: material modeling; (c) void growth: growth mechanisms; and (d) void growth: void geometry. These are exemplified by the Cockcroft-Latham^27^, the Gurson-Tvergaard-Needleman^28^,^29^, and the continuum damage mechanics (CDM) or CDM-based^30^,^31^,^32^ fracture criteria. In addition, the micromechanics of ductile fracture have made enormous progress in recent years^33^. All the above models assume that the fracture evolution directly depends on the strength, strain hardening capability and strain rate sensitivity of the matrix surrounding the voids. None of these criteria consider the change in microstructure during ductile fracture.

In this paper, we try to answer the question whether the ductile fracture behavior of metals is only determined by the voids. Ultrafine-grained Al-Mg alloy sheets were fabricated by cold rolling, AR, cryorolling, and ACR respectively. The ductility of the sheets produced using all four rolling techniques reduces as the number of rolling passes increases, while the grains are refined gradually. The mean grain size before and after fracture at the shear band zone were examined. We found that the mean grain size near the fracture surface is much smaller than that in the matrix, and is close to the theoretical achievable minimum.

## Results

**Figure 1:**
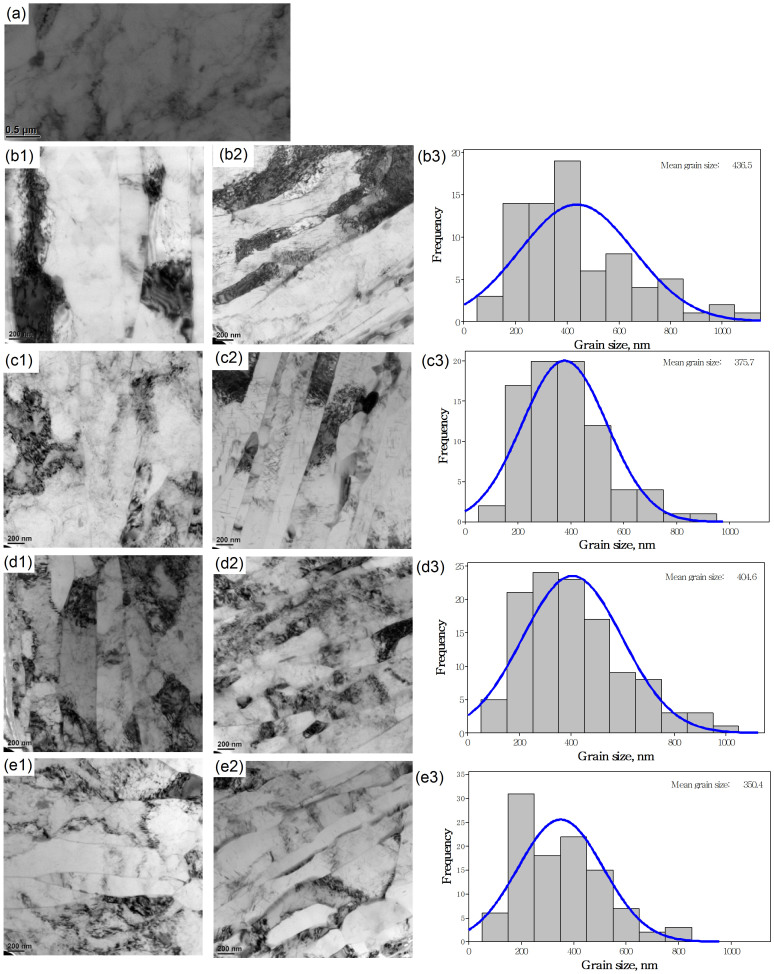
TEM images of rolled sheets under different rolling techniques. (a) annealed sample, (b1) and (b2) after the first and third pass of cold rolling, (b3) grain size distribution after the third cold rolling pass; (c1) and (c2) after the first and third pass of AR, (c3) grain size distribution after the third AR pass; (d1) and (d2) after the first and third pass of cryorolling, (d3) grain size distribution after the third cryorolling pass; (e1) and (e2) after the first and third pass of ACR, (e3) grain size distribution after the third ACR pass.

Fig. 1a shows the coarse-grained microstructure of annealed samples used in the rolling process. Fig. 1b1 and Fig. 1b2 show the microstructures of sheets produced using cold rolling after the first and third pass respectively. As the number of rolling passes increases, the coarse grains are progressively refined. Fig. 1b3 shows the grain size distribution after the third cold rolling pass, when the mean grain size is 436.5 nm. Fig. 1c1 and Fig. 1c2 show the microstructures of sheets produced using AR after the first and third pass. Both the grain sizes are smaller than those shown in Fig. 1b. After the third AR pass, the mean grain size has been refined to 375 nm, as shown in Fig. 1c3. Fig. 1d1 and Fig. 1d2 show the microstructures of sheets produced using cryorolling after the first and third pass. The mean grain size induced by cryorolling is much smaller than that by conventional cold rolling, and it is slightly larger than that due to AR, as shown in Fig. 1d3. Fig. 1e1 and Fig. 1e2 show the microstructures of sheets fabricated using ACR. The mean grain size of 350 nm obtained after the third ACR pass is much smaller than that obtained by the other three processes.

**Figure 2:**
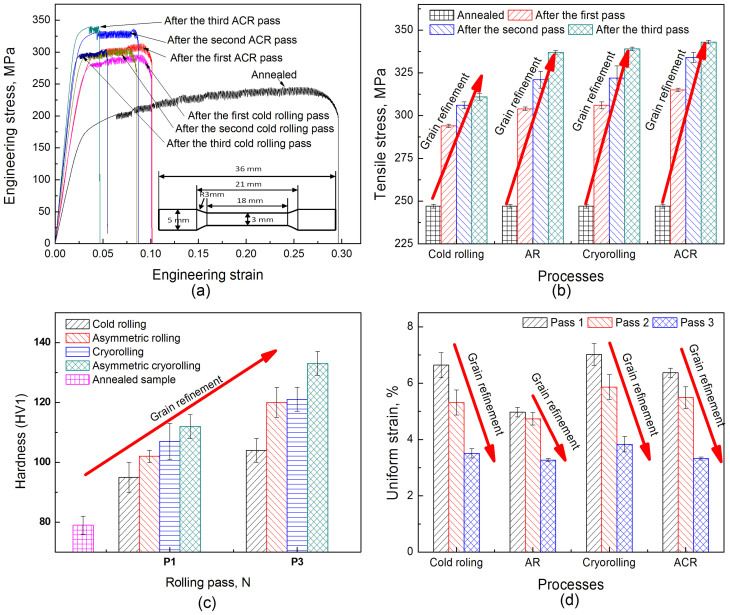
Mechanical properties of sheets under different rolling techniques. (a) Engineering strain vs. engineering stress; (b) Tensile stress vs number of rolling passes; (c) Hardness vs number of rolling passes; (c) Uniform strain vs number of rolling passes.

Fig. 2 shows the mechanical properties of the rolled sheets. Fig. 2a presents the engineering stress vs engineering strain curve for cold-rolled samples and ACR-processed samples. It can be seen that serrated deformation occurs during the tensile test. Lüders strain is an inherent feature of Al-Mg alloys with fine grains. This is attributed to the dynamic strain-ageing effect, and to interactions between the mobile Mg solute atoms and dislocations^34^. Zhao *et al.*^35^ pointed out that the critical strain *ε_c_* for initiation of serrated flow increases considerably with increasing strain and Mg content, contrary to the behavior of coarse-grained and non-deformed counterparts. Fig. 2b shows that the tensile stress of sheets increases for these four rolling techniques as the number of rolling passes increases. In addition, the tensile stresses of samples produced using cryorolling and AR have similar values, higher than those produced using cold rolling, but lower than those produced using ACR. Fig. 2c, shows that the changes in sample hardness follow similar trends. With grain refinement, the hardness of the samples increases. Generally for metals, the yield stress is about three times the hardness^19^.

It is well known that the dislocations in crystalline solids are thermodynamically unstable entities^36^. Low temperatures can lead to a higher dislocation density that can effectively suppress dynamic recovery. At cryogenic temperatures, the suppression of dynamic recovery during deformation is expected to preserve the high density of defects generated by deformation^37^. It is easy to understand why, with the same strain, the grains refined at cryogenic temperatures are smaller than those at room temperature. In Fig. 1, the mean grain size of sheets produced by cryorolling is seen to be 404 nm, compared to 436 nm for cold rolling. The mean grain size of sheets produced using ACR is 350 nm, compared to 375 nm for sheets produced using AR. According to the Hall-Petch relationship, 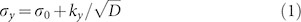
where *σ_y_* is the yield stress after deformation, *σ*_0_ is the yield stress after annealing, *k_y_* is the Petch parameter, and *D* is the mean grain size, the yield stress, hardness and tensile stress of sheets processed at cryogenic temperatures are greater than those processed at room temperature, as shown in Fig. 2b and Fig. 2c. Due to the minimum mean grain size of sheets produced using ACR, the hardness, yield stress and tensile stress are higher than in sheets produced using the other three processes.

Fig. 2d shows the uniform strain of sheets as a function of the number of passes for various processes. The uniform strain of the sheets has been widely used to analyze the ductility of nano-grained sheets/foils^38^. As the number of rolling passes increases, the ductility of the sheets reduces for the four rolling techniques. The figure also shows that the samples produced using cryorolling have the highest uniform strain, and those produced using AR have the lowest uniform strain. It is also very interesting that the uniform strain resulted from cryorolling is higher than that resulted from cold rolling, and that the uniform strain produced using ACR is higher than that produced using AR. This implies that the ductility of Al-Mg alloys deformed at cryogenic temperatures is higher than that of the material deformed at room temperature.

**Figure 3:**
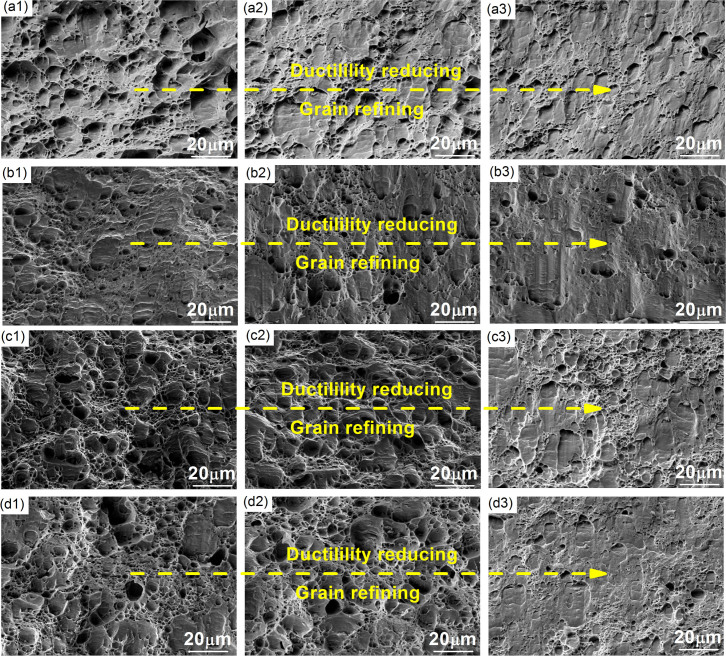
SEM images of the fracture surface of tensile samples after (a1) the first cold rolling pass, (a2) the second pass, (a3) the third pass; (b1) the first AR pass, (b2) the second pass, (b3) the third pass; (c1) the first cryorolling pass, (c2) the second pass, (c3) the third pass; (d1) the first ACR pass, (d2) the second pass and (d3) the third pass.

Fig. 3 shows sequentially the SEM images of fracture surfaces. In the Figure, ‘1’ stands for the first pass, ‘2’ the second pass, and ‘3’ the third pass. As the number of rolling passes increases, the dimples on the fracture surfaces gradually reduce and become shallower. This means that the ductility reduces as the mean grain size decreases, as shown in Fig. 2d. In Figs. 3a3 to d3 reveal the occurrence of cleavage fracture at some locations. In addition, the dimples on the fracture surface are both deeper and more in number for cryorolled samples than for cold-rolled samples. Similarly, the dimples on the fracture surface of ACR-processed samples are both deeper and more in number than for AR-processed samples.

## Discussion

**Figure 4:**
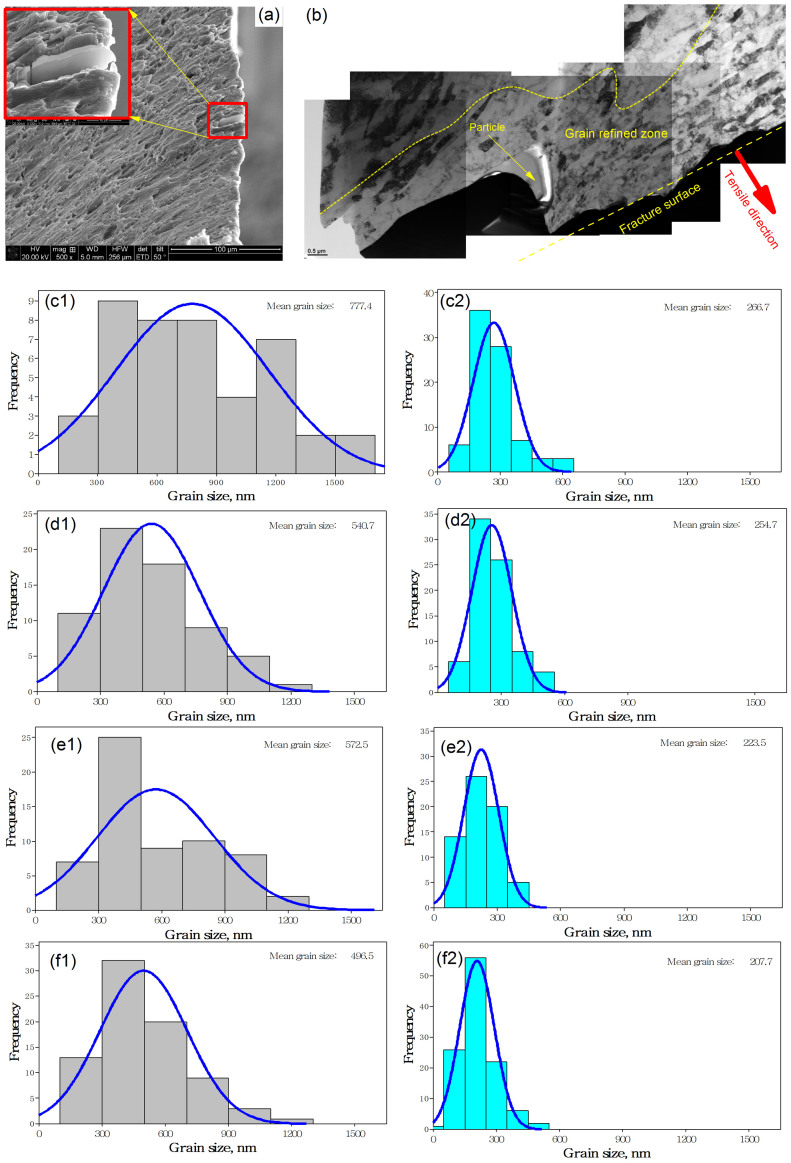
(a) SEM of a FIB sample on the fracture surface; (b) TEM images of microstructure near fracture surface for cryorolling; (c1) ~ (f2), grain size distribution of samples. (c1) grain size of samples after cold rolling and (c2) after tensile test; (d1) grain size of samples after AR and (d2) after tensile test; (e1) grain size of samples after cryorolling and (e2) after tensile test; (f1) grain size of samples after ACR and (f2) after tensile test.

In Fig. 2d and Fig. 3, we observed that the ductility of the sheets reduces with grain refinement for all four rolling techniques. This observation suggests that the ductile fracture of the sheets may be determined by the grain evolution in the shear banding zone, apart from the void growth mechanisms. Here we propose close examination of the change in microstructure at the fracture site. Fig. 4a shows a detail of the SEM image of a focused ion beam (FIB) sample at the fracture surface of a tensile test sample. Fig. 4b (also Figs. S1, S2, S3 and S4) shows the microstructure near the fracture surface of the samples after the first cryorolling pass. The figure shows that there are some voids, cracks and inclusions. Generally these features characterize ductile fracture. However, they only occupy a small part of the full image. In addition, the processes were carried out on the same sheets, so that there is no difference in their inclusion content. The rolling process may affect the stress state and the initiation of microcracks around the inclusions^39^. The inclusions are very likely to be more brittle and cracks would form more easily during deformation at cryogenic temperatures. However, the ductility of the sheets deformed at cryogenic temperature is greater than those deformed at room temperature. Thus, in the current study, the influence of deformation of inclusions on the ductility can be neglected. In Fig. 4b, we noticed another interesting feature of the local grain size distribution near the fracture surface. The grains near the fracture surfaces are refined. Figs. 4c1 to 4f2 show the grain size distribution before and after tensile processing. After the tensile test, the grains in the local zone near the fracture surface are more refined than before the tensile test. For the samples produced using cold rolling, the mean grain size is reduced from 777 nm to 266 nm. For AR, cryorolling and ACR, the corresponding mean grain size is reduced from 540 nm to 254 nm, 572 nm to 223 nm, and 496 nm to 207 nm respectively. For the Al-Mg alloy, the minimum mean grain size is given by the following equation^40^:
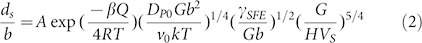
where *d_s_* is the steady-state grain size, *b* is the Burgers vector, *A* is a constant, β ≈ 0.04, *Q* is the activation energy for self-diffusion, *R* is the universal gas constant, *T* is the absolute temperature, *D_PO_* is the frequency factor for pipe diffusion, *G* is the shear modulus, *v*_0_ is the initial dislocation velocity, *k* is Boltzmann's constant, *γ_SFE_* is the stacking fault energy, and *HV_S_* is the steady-state hardness. According to the theoretical calculation by Edalati *et al*^40^, the minimum achievable mean grain size of an SPD-processed Al-Mg alloy at room temperature is around 230 ~ 330 nm. It is obvious from Fig. 4 that the mean grain size of sheets at fracture approaches the theoretical minimum value (see Fig. S5).

During deformation, grains are often refined as a consequence of fracture in ductile materials. It is known that nano-grained metals often have reduced ductility. For a coarse-grained Cu sample, the uniform elongation reaches 31 ± 2%, however a free-standing nano-grained Cu foil showed a uniform elongation of <2%^41^. During the tensile test, the grains undergo significant local refinement, causing embrittlement, until the occurrence of failure. There are a number of criteria associated with ductile fracture^26^,^27^,^28^,^29^,^30^,^31^,^32^. Void nucleation and growth play a key role leading to necking. Some of them assume that the materials will fail when the void volume fraction reaches a certain value. However, none of those criteria consider the effect of grain refinement during plastic deformation post necking that is often observed in shear band zone^42^ and as the crack propagates along the shear band zone^43^. Among these fracture criteria, the Cockcroft-Latham fracture criterion is widely used in engineering analysis. The Cockcroft-Latham fracture criterion assumes that the maximum principal stress is the most relevant in the initiation of fracture. The criterion is defined in terms of traction plastic work associated with the principal stress along the path of the equivalent plastic strain, as shown in following equation: 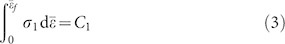
where σ_1_ is the maximum principal stress; 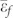 is the critical plastic strain at fracture; and *C*_1_ is the critical damage value at fracture.

**Figure 5:**
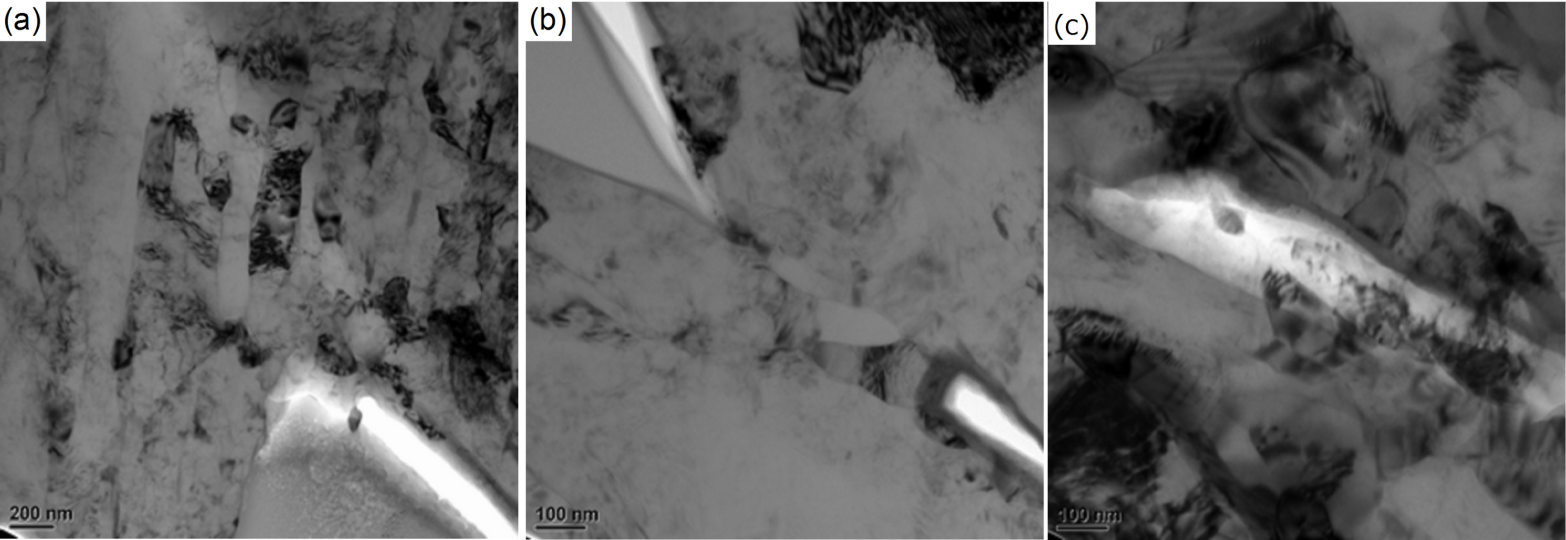
TEM images of refined grains at the (a) inclusion, (b) crack tip and (c) void.

Based on the Hall-Petch relationship and taking into account arguments involving the relationship between work hardening and dislocation density, the dependence of the flow stress on strain can be given by^44^:
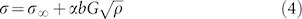
where *σ_∞_* is the friction stress, *G* is the shear modulus, *b* is the Burgers vector and *α* is a constant. In the case of severe plastic deformation, the generated dislocation density is much greater than the original dislocation density. Here we assume that the dislocation density in metals during deformation nearly equals the generated dislocation density. Thus the dislocation density *ρ* can be calculated in terms of the strain using^45^: 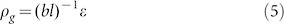
where *ρ_g_* is the total generated dislocation density, *b* is the Burgers vector, *l* is the mean free path for the dislocation movement, and *ε* is the strain. From the equations (1) ~ (5), we can obtain the relationship between mean grain size and strain:
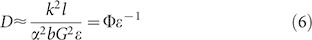
where 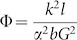, can be assumed to be a constant for a material. This equation shows that the mean grain size is inversely proportional to the strain. When the strain increases to the critical plastic strain at fracture, the mean grain size will be reduced to a minimum value:
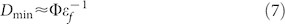
In addition, as the mean grain size reduces, the metal becomes brittle. For brittle materials, generally the fracture stress equals the yield stress. From Eq. (1) and Eq. (3), the fracture criterion could be revised as:
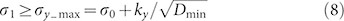
Eq. (8) shows the relationship between fracture and the mean grain size. During deformation, grains are often refined to the minimum size in the shear band zone. This may well be the direct mechanism for fracture in ductile materials post necking. We have known that the nano-grained metals often have low ductility. During the tensile process, the grains in the shear band zone undergo severe local refinement, become more and more brittle until finally failure occurs. As shown in Fig. 5a, when the crack initiation occurs between an inclusion and the matrix, the grains in the matrix are very small. In Fig. 5b, it is seen that when a crack propagates, the grains at the crack tip are also very small. As seen in Fig. 5c, when a void expands, the grains around the void are refined.

**Figure 6:**
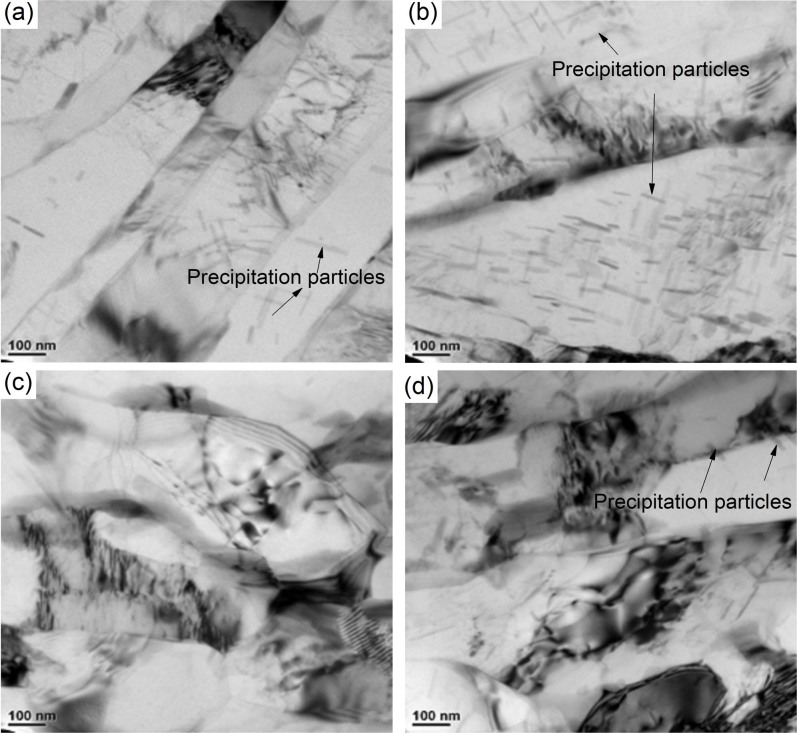
Precipitation particle distribution in sheets after the third pass: (a) cold rolling, (b) AR, (c) cryorolling, (d) ACR.

Greater strength and toughness are desirable for most structural materials. However, for some nano-grained metals, generally ductility and strength are conflicting properties^25^. As shown in Fig. 1 and Fig. 2, the ductility of the sheets gradually reduces with reduction in grain size. It is very important to improve the ductility of nano-grained metals, and generally, there are four ways of achieving this: (i) reducing the stacking fault energy and increasing the solute content^46^; (ii) application of nanotwins^47^; (iii) application of gradients structure^48^,^49^; and (iv) controlling the grain size distribution^17^. For nano-grained copper sheets produced using cryorolling, Wang *et al.*^17^ believed that an inhomogeneous microstructure stabilized by strain hardening mechanisms, tensile deformation leads to a high tensile ductility. However, except for the smaller grain size resulted from rolling at cryogenic temperatures, we do not find any difference in grain size distribution in Fig. 1a and Fig. 1c, and in Fig. 1b and Fig. 1d. Therefore this mechanism cannot adequately explain the improvement in ductility of these Al-Mg sheets deformed at cryogenic temperature. In Fig. 4, the mean grain size of sheets produced at cryogenic temperature after fracture is seen to be smaller than that of those produced at room temperature, and this may explain the improvement in ductility. A related question is: why do the Al-Mg sheets processed at cryogenic temperatures attain a smaller mean grain size at the fracture surface? The stacking fault energy on the equilibrium grain size affects the tensile properties of nano-grained metals. Huang *et al.*^50^ reported that with increasing solute content and decreasing stacking fault energy, not only the strength but also the ductility of nano-grained copper alloys increased substantially. For the Al-Mg alloy, the distribution of Mg in Al will directly affect the mechanical properties of the sheets. Fig. 6 shows the microstructure of the sheets after the third pass using different processes. It is seen that when the sheets were rolled at cryogenic temperature, there are only few precipitation particles, much fewer than for the sheets rolled at room temperature. The suppression of large precipitation particles in the sheets will result in a higher Mg solute content in the Al matrix. The smaller the precipitation particles, the more refined would the grains be during the tensile fracture. This will directly improve the ductility of the sheets as shown for nanoparticle composites^51^,^52^.

In summary, Al-Mg alloy sheets were fabricated using four different techniques: cold rolling, asymmetric rolling, cryorolling and asymmetric cryorolling. As the number of rolling passes increases, the ductility of the sheets as well as the mean grain sizes reduces. A new mechanism was proposed to explain the ductile fracture of Al-Mg sheets post necking during tensile tests at low strain rate. The proposed mechanism includes other influences besides the voids. The sequence of steps leading to ductile fracture are: uniform elongation, void nucleation and growth, local necking, grain refinement until the mean grain size approaches the minimum achievable mean grain size, and finally damage. During the tensile process, the grains in the sheets produced at cryogenic temperature could be refined to a smaller size compared with those produced at room temperature. This may also explain the improvement in the ductility of the sheets at cryogenic temperatures. In addition, electron backscatter diffraction (EBSD) study on the microstructure near fracture surface should be carried out in the future^53^.

## Methods

In the experiments, 1 mm thick Al-Mg alloy (AA5052) sheets were used. Their chemical composition was Mg-2.5, Si-0.25, Fe-0.4, Cr-0.15, Zn-0.1, Ti-0.94, Cu-0.1, Mn-0.01, and Al-balance. Al-Mg alloys possess excellent properties such as high corrosion resistance, high strength to weight ratio, weldability and formability. They have therefore been widely employed as structural materials in industrial applications such as aerospace, automotive, marine and other industries. A 4-high multifunction rolling mill was used to carry out the rolling experiments. Different original sheets were subjected to four kinds of process: cold rolling, AR, cryorolling, and ACR. The rolling speed ratio between the up and bottom rolls was 1.4 for AR and ACR. For cryorolling and ACR, the sheets were dipped in liquid nitrogen (−196°C) for at least 8 minutes^19^, satisfying the requirement that the temperature of the sheets should be lower than −100°C after each pass^17^. The thicknesses of the rolled sheets after each successive pass were 0.6 mm, 0.4 mm and 0.2 mm for cold rolling, AR, cryorolling and ACR respectively. Tensile tests with a strain rate of 1.0 × 10^−3^ s^−1^ on an INSTRON machine were used to determine the engineering stress vs engineering strain characteristics. The tensile tests were repeated three times for each thickness. An FEI xT Nova Nanolab 200 Dual-beam workstation was used to prepare thin foil specimens from the rolled and tensile samples for TEM observation. A Philips CM200 field emission gun transmission electron microscope (FEG/TEM) equipped with a Bruker Energy Dispersive X-ray (EDAX) spectroscopy system operating at an accelerating voltage of 200 kV was used to investigate the details of the microstructure. For the severe plastic deformation processed metals, the TEM technique is widely used to study the grain size and distribution^54^,^55^.

## Author Contributions

Y.H. conceived the study. Y.H., T.A.K. and L.C. wrote the main manuscript text. Y.H., L.X., L.M. and K.C. conducted the experiments. Y.H., T.A.K., L.C., G.A. and Q.Q. analyzed the data. All authors reviewed the manuscript.

## Supplementary Material

Supplementary Information Supplementary Information
