# Supplementary material for: A new insight into ductile fracture of ultrafine-grained Al-Mg alloys
Source: Sci Rep. 2015 Apr 8;5:9568. doi: 10.1038/srep09568 (PMC4389191; doi:10.1038/srep09568)
Supplement: Supplementary Information [file srep09568-s1.doc]

**A new insight into ductile fracture of ultrafine-grained Al-Mg alloys**

Hailiang Yu1[[1]](#footnote-2), A. Kiet Tieu^1^, Cheng Lu^1^, Xiong Liu^1^, Mao Liu^1^, Ajit Godbole^1^, Charlie Kong^2^ & Qinghua Qin^3^


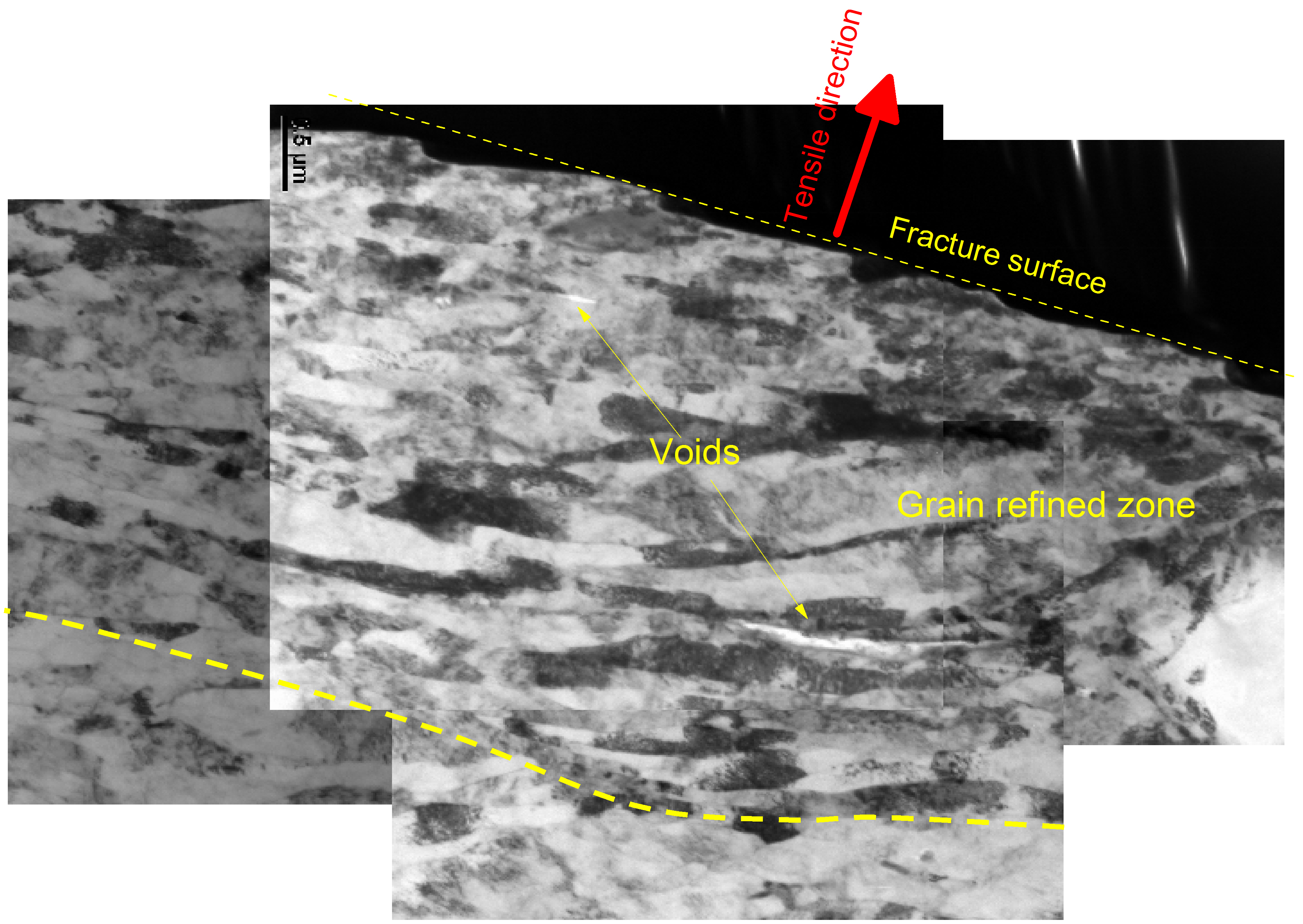


**Figure S1 | TEM images of microstructure near fracture surface of sheets produced using cold rolling.**


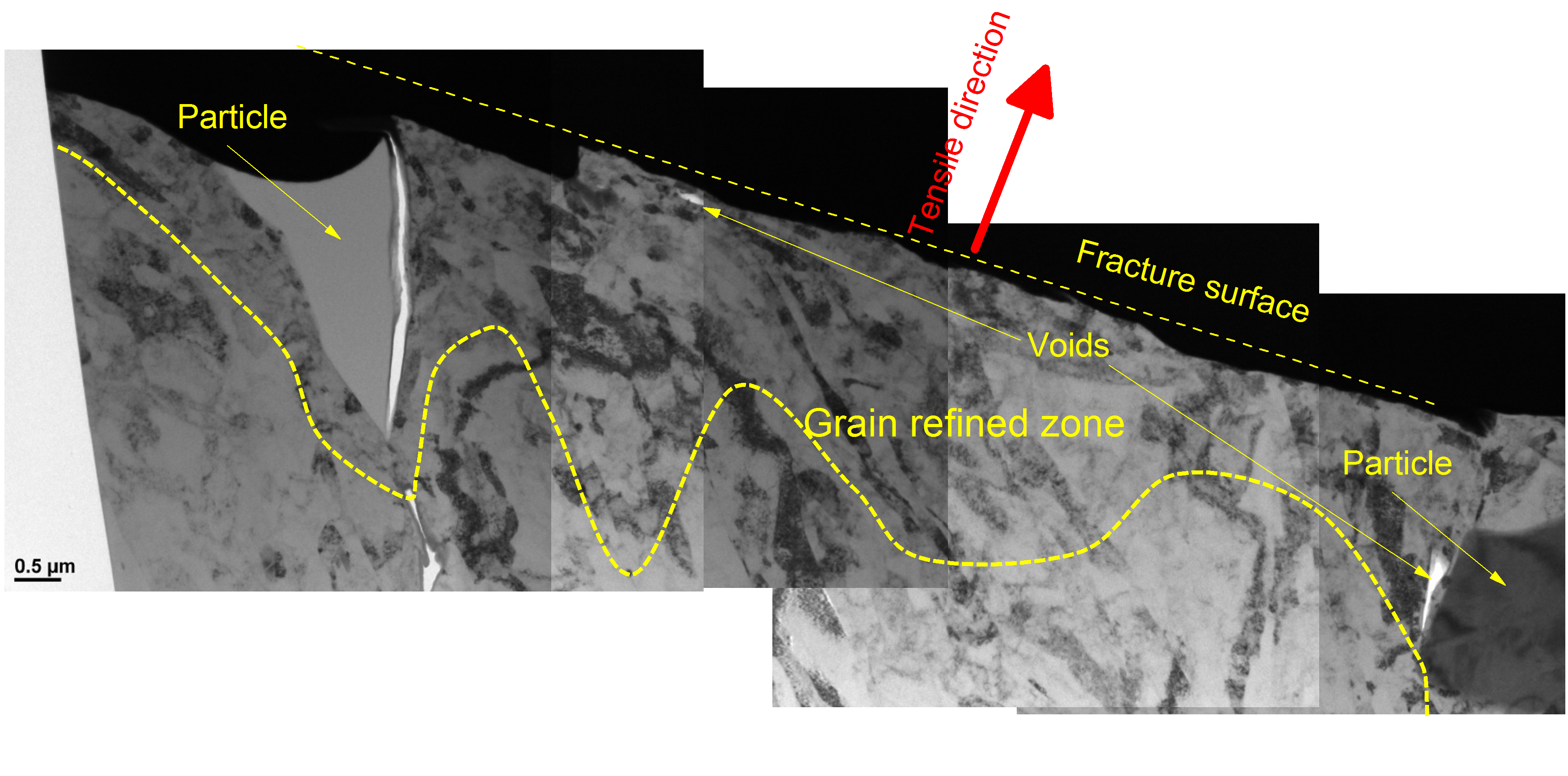


**Figure S2 | TEM images of microstructure near fracture surface of sheets produced using asymmetric rolling.**


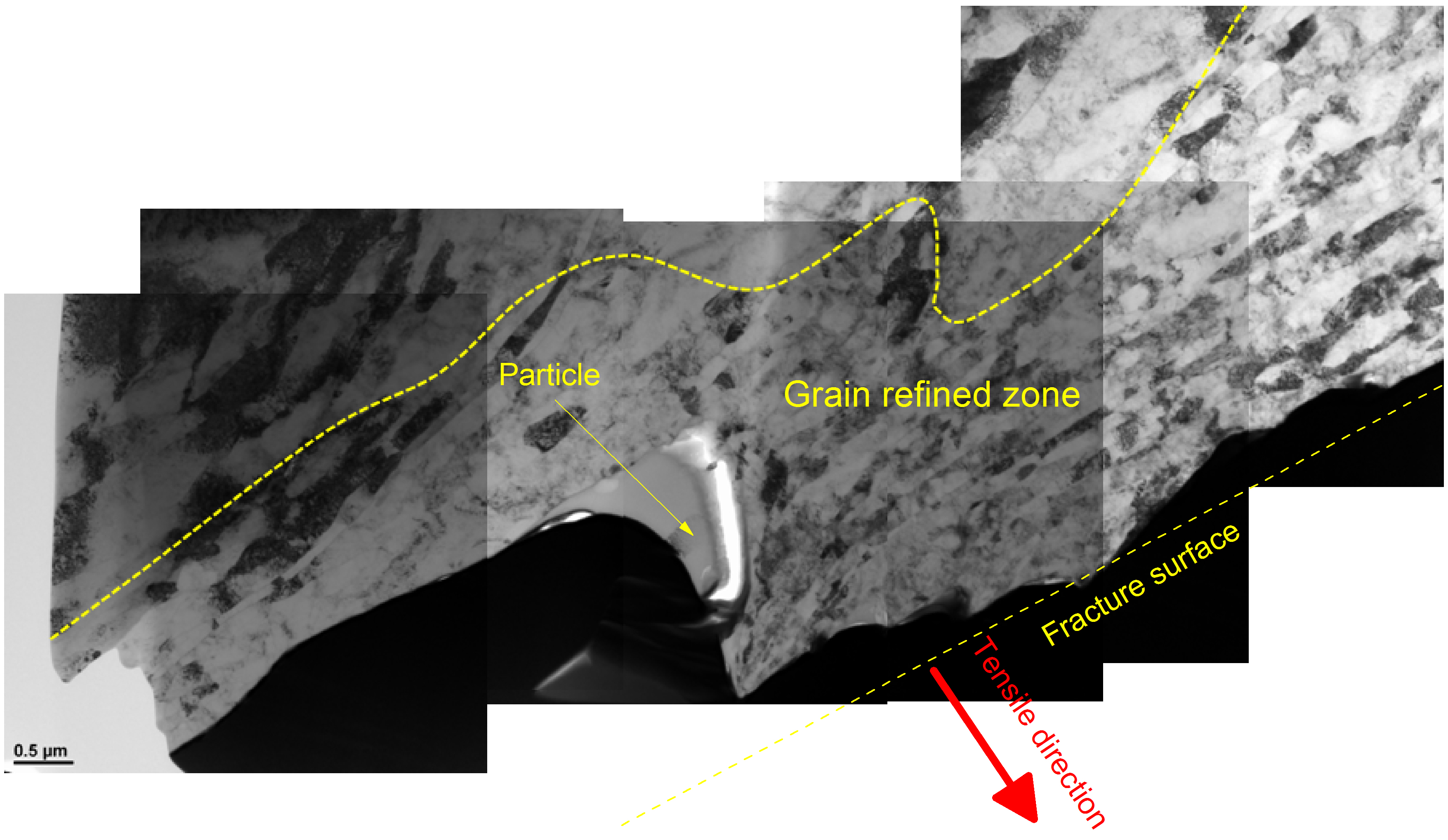


**Figure S3 | TEM images of microstructure near fracture surface of sheets produced using cryorolling.**


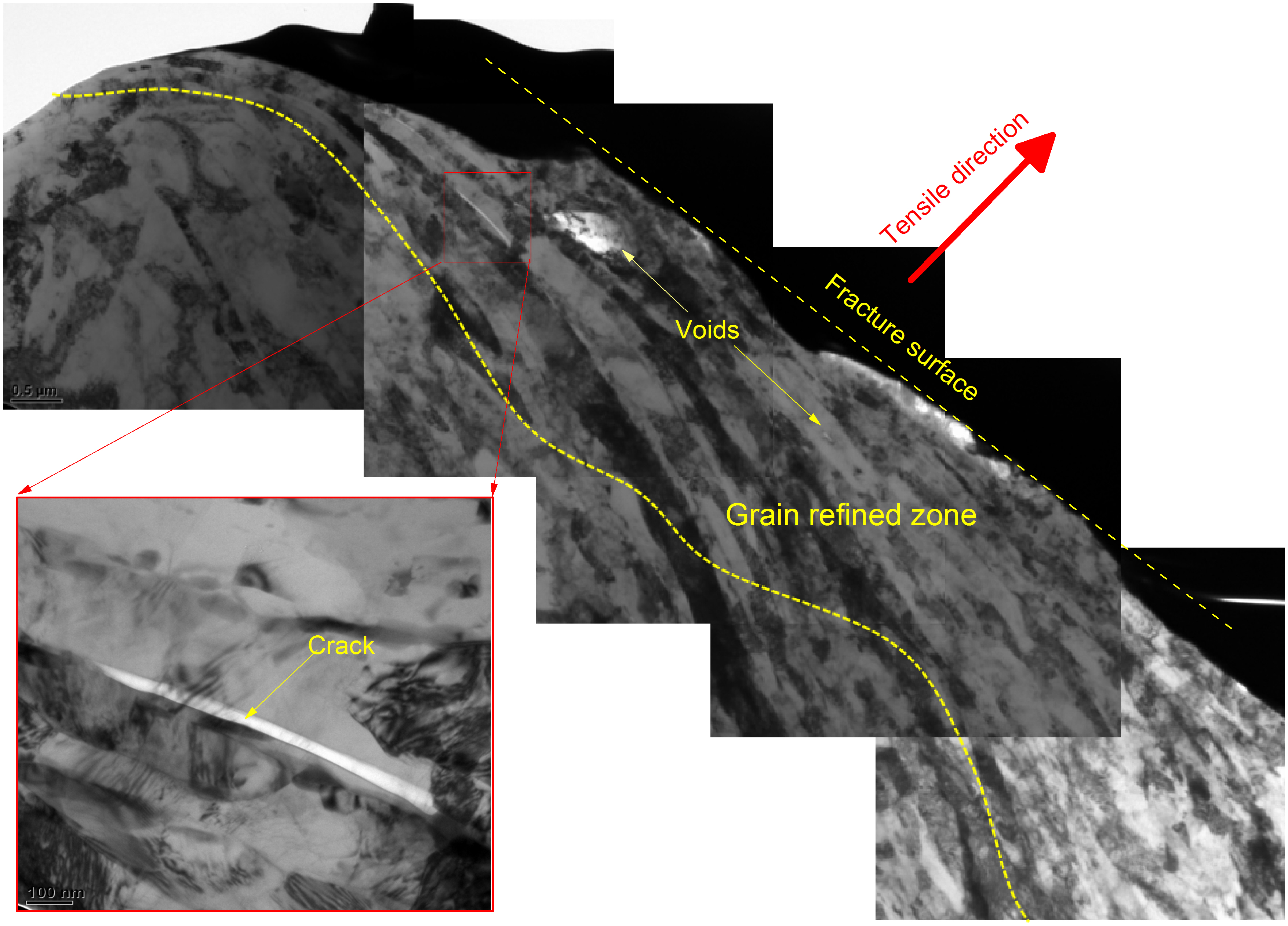


**Figure S4 | TEM images of microstructure near fracture surface of sheets produced using asymmetric cryorolling.**


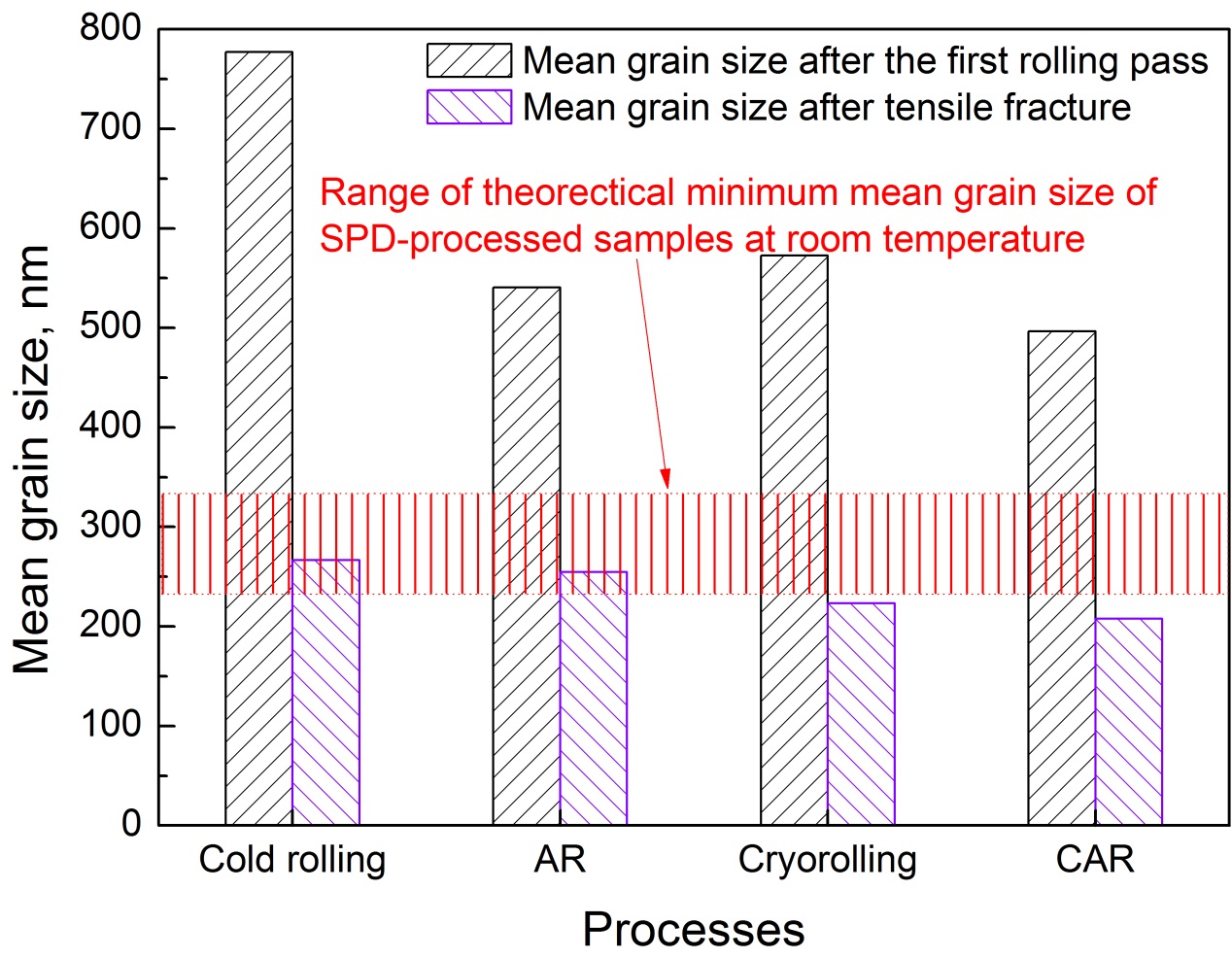


**Figure S5 | Comparison of mean grain size after rolling processes and tensile test.**

1. Correspond author: Hailiang Yu, email: [hailiang@uow.edu.au](mailto:hailiang@uow.edu.au); [yuhailiang1980@gmail.com](mailto:yuhailiang1980@gmail.com) [↑](#footnote-ref-2)
